# Transcatheter closure of an atrial septal defect with high risk of erosion using a Figulla Flex II atrial septal defect occluder

**DOI:** 10.1007/s12928-017-0457-x

**Published:** 2017-01-20

**Authors:** Norio Tada, Yukiko Mizutani, Takashi Matsumoto, Mie Sakurai, Tatsushi Ootomo

**Affiliations:** Department of Cardiology, Sendai Kousei Hospital, 4-15 Hirosemachi, Aoba, Sendai, Miyagi 980-0873 Japan

**Keywords:** Atrial septal defect, Figulla Flex II ASD occluder

## Abstract

An 85-year-old man with a high risk for open heart surgery underwent a percutaneous closure of an atrial septal defect that lacked adequate aortic and superior rims. To avoid the risk for erosion, a Figulla Flex II ASD occluder was selected for the procedure. Implantation was successful, and no complications were observed during the 6 months of follow-up.

## Introduction

The Amplatzer septal occluder (ASO) is the most widely used device in the transcatheter closure of atrial septal defects (ASDs). Cardiac erosion is a rare; however, severe complications occur in 0.2% of patients [1, 2]. The risk for erosion has been associated with oversized devices and an inadequate aortic rim [2]. Because the Figulla Flex II ASD occluder (FFO, Occlutech GmbH, Jena, Germany) disc has a softer edge compared with other devices, it may reduce the risk for erosion. We present a case of transcatheter ASD closure using FFO in a patient with a high morphological risk for erosion.

## Case

**Fig. 1:**
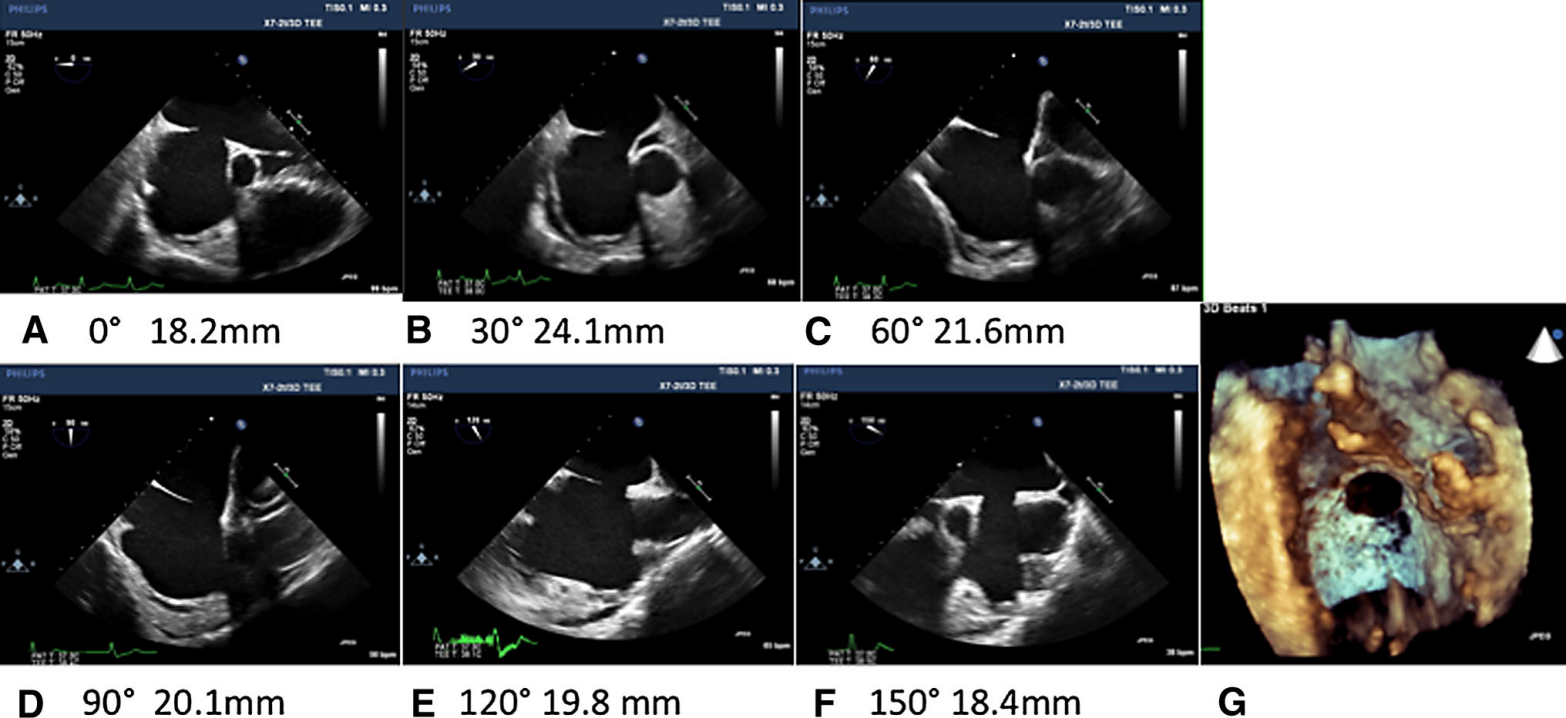
Transesophageal echocardiography of the atrial septal defect. Two-dimensional images and defect diameters each at 30° (**a**–**f**) and a three-dimensional right atrial en-face view image (**g**) are shown. The maximum defect diameter was 24.1 mm at 30°, and the minimum defect diameter was 18.2 mm at 0°. The aortic rim was deficient at 30°–60°, and the superior rim was deficient at 60°–90°. The posterior rim was not floppy and was sufficiently long. Septal malalignment was observed at 60° and 90°

An 85-year-old man with a history of recurrent hospitalization because of congestive heart failure with pulmonary hypertension, chronic atrial fibrillation, and chronic kidney disease was referred to our hospital for ASD closure. Transthoracic echocardiography (TTE) confirmed a secundum ASD, right ventricular enlargement, and calculated shunt ratio (*Q*_p_/*Q*_s_) of 1.8. Transesophageal echocardiography (TEE) was performed for a morphological assessment (Fig. 1). The maximum diameter of the defect was 24.1 mm at 30°, and the minimum diameter of the defect was 18.2 mm at 0°. The aortic rim was deficient at 30°–60°, and the superior rim was deficient at 60°–90°. The posterior rim was not floppy and was sufficiently long. Septal malalignment was observed at 60° and 90°. We assessed the patient as being at a high risk for erosion or migration of the closure device because of rim deficiency and septal malalignment. He still complained of shortness of breath on exertion under medical treatment. Because his history also placed him at a high risk for open heart surgery, we chose to perform a transcatheter closure using FFO. Written informed consent was obtained from the patient and his family prior to the procedure.

## Procedure

**Fig. 2:**
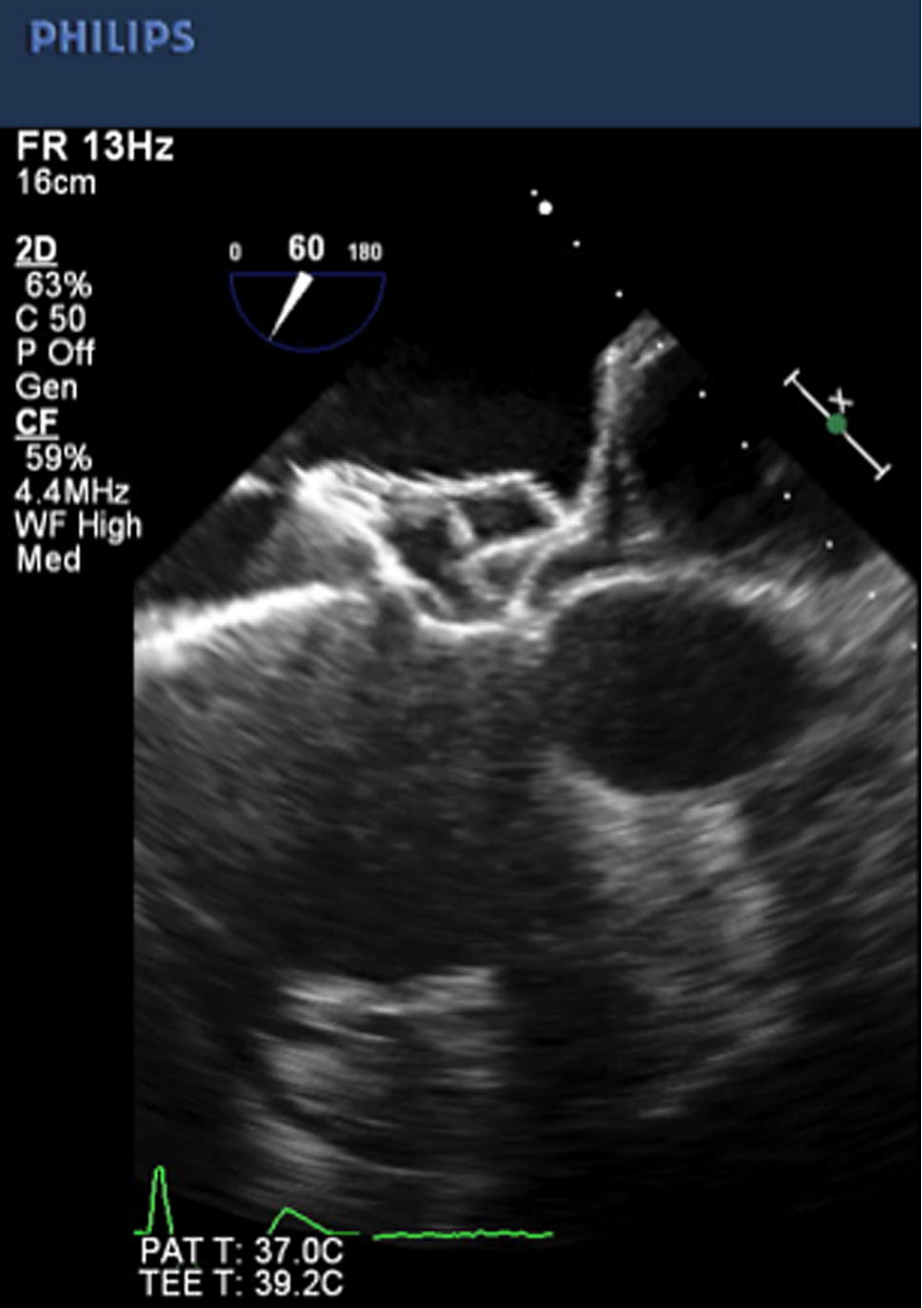
Transesophageal echocardiographic image after deploying the Figulla Flex II ASD occluder. The cable remains connected. A ball-shaped connector design allows a tilt of up to 50°, facilitating placement before release

**Fig. 3:**
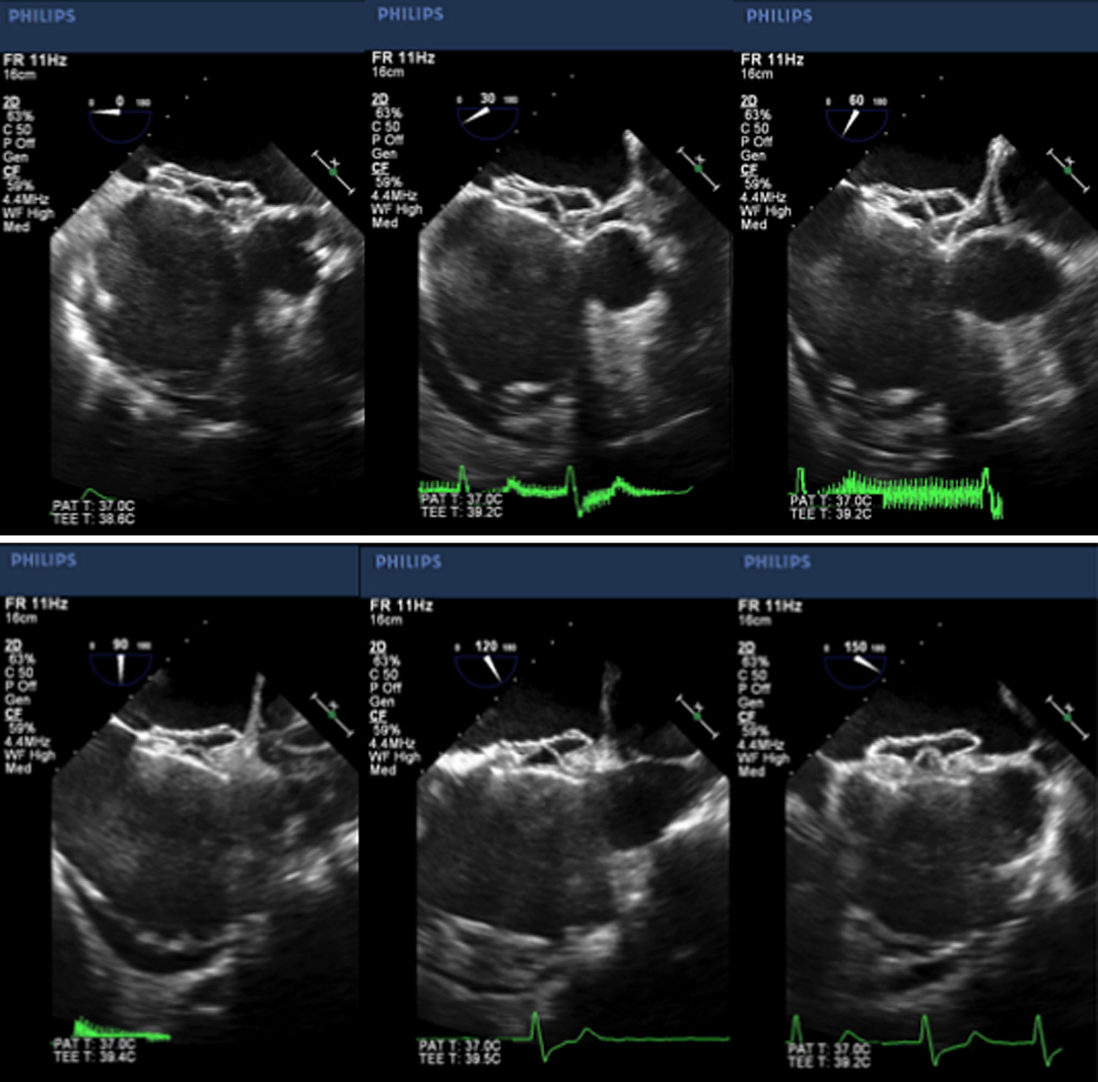
Transesophageal echocardiographic (TEE) image after deploying the Figulla Flex II ASD occluder. The device was stable, and TEE at 30° and 60° views showed good device conformability with the malaligned septum and minimal left disc tenting of the atrial free wall into the transverse sinus

Percutaneous ASD closure was performed under TEE guidance. Balloon sizing was measured to be 27 mm. The FFO size could be 27 or 30 mm because the maximum diameter of the defect was 24.1 mm. Considering the oval shape with a minimum diameter of 18.2 mm and aortic and superior rim deficiency, we chose and deployed a 27-mm FFO (Fig. 2). A ball-shaped connector design allows a tilt of up to 50°, facilitating the assessment of deployment. After release, the device was stable and TEE at 30° and 60° views showed good device conformability with the misaligned septum and minimal left disc tenting of the atrial free wall into the transverse sinus (Fig. 3). The procedure was complete.

## Follow-up

**Fig. 4:**
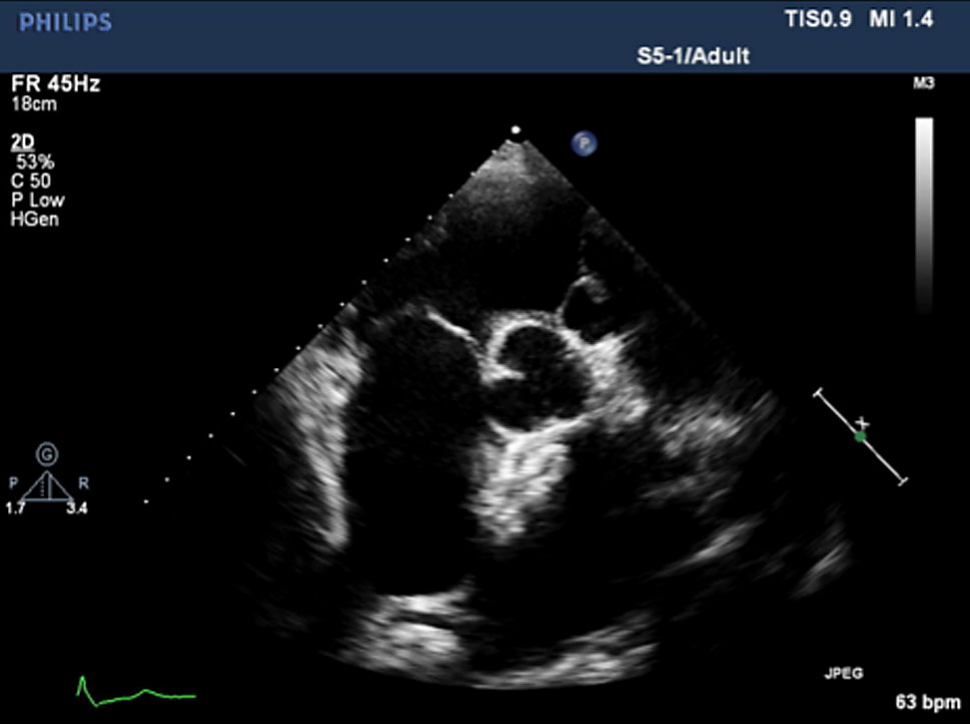
Transthoracic echocardiographic short axis view after 6 months of follow-up. The device contacts the sinus of Valsalva smoothly, and the pericardial effusion has not increased

Patient recovery was uneventful with NYHA 1 during the 6 months of follow-up. TTE revealed adequate sandwiching of the septum and smooth contact with the sinus of Valsalva (Fig. 4). Pericardial effusion did not increase.

## Discussion

Erosion is a rare but severe complication of transcatheter ASD closure [1, 2]. Aortic rim deficiency and implantation of an oversized device are risk factors for erosion [2]. Amin [3] reported that echocardiographic evidence of aortic rim absence in multiple views, poor posterior rim consistency, septal malalignment, and dynamic changes in ASD indicated an increased risk for erosion. Evidence of device tenting of the atrial free wall into the transverse sinus, as in this case, was also a risk factor [3]. The morphological characteristics of this patient were consequently indicated as a high risk for erosion using ASO.

FFO is a double-disc occluder that is similar to ASO, but its left disc utilizes a unique braiding system without a hub, reducing the amount of material and giving it a lower profile than ASO. This construction results in a softer contact with aortic vessel walls if placed adjacent to it [4]. Erosion has not yet been reported in cases wherein FFO was used [4–6]. Haas et al. [4] reported a registry of Occlutech device use for 1315 patients. There were no cases of erosion in the registry. According to device embolization, 1.1% occurred during implantation and 0.4% during follow-up. Most defects had no rim, and they clearly demonstrated an increased risk for device embolization when balloon sizing was not performed. Therefore, we still require discussions regarding the interaction differences of devices and the risk for embolization. However, during deployment, the left atrial portion has a round, ball-like shape unlike the flat profile of devices with a double-sided hub. This prevents a prolapse of the left disc during implantation, particularly in large ASDs, those without a rim, or those with a minimal aortic rim [5].

Six months after the procedure, the patient had not experienced any complications. Because a late occurrence of erosion or migration has been reported with ASO, we need to continue close follow-up [7–9]. FFO may be a solution for patients with a morphologically high risk for transcatheter ASD occlusion.
